# Pregnancy Loss Was Associated With the Increased Risk of Cardiovascular Diseases in Middle-Aged Women: Evidence From the China Health and Retirement Longitudinal Study

**DOI:** 10.5334/gh.1386

**Published:** 2025-01-09

**Authors:** Xiaoyan Yang, Qingling Fan, Can Shen, Ruirui Hou, Ruoling Chen, Jiaqian Yin, Huifeng Xiang, Yunxia Cao, Xiaoqing Peng

**Affiliations:** 1 Department of Obstetrics and Gynecology, the First Affiliated Hospital of Anhui Medical University, Hefei, Anhui, China; 2 NHC Key Laboratory of Study on Abnormal Gametes and Reproductive Tract (Anhui Medical University), Hefei, Anhui, China; 3 School of Pharmacology, Anhui Medical University, Hefei, Anhui, China; 4 Faculty of Education, Health and Wellbeing, University of Wolverhampton, Wolverhampton, UK

**Keywords:** induced abortion, miscarriage, coronary heart disease, stroke, reproductive health

## Abstract

**Objectives:**

Significant associations between pregnancy loss and risk of future maternal cardiovascular disease (CVD) have been found in Western countries, but the association in China is still unclear. Therefore, this study aimed to investigate the associations of pregnancy loss, number of pregnancy losses, subtype of pregnancy loss (i.e. induced abortion, miscarriage and stillbirth) and age at the first pregnancy loss with CVD risk in Chinese population.

**Methods:**

We examined data of 7,486 middle-aged women (mean age 58.1 years) from the China Health and Retirement Longitudinal Study. Pregnancy loss and CVD including coronary heart disease (CHD) and stroke were self-reported and documented in surveys.

**Results:**

In the cohort, 1,850 (24.7%) women experienced pregnancy loss. Over 39 years follow-up, 2,055 (27.5%) women developed CVD. After adjusting covariates, pregnancy loss was associated with the risk of CVD (HR 1.73, 95% CI 1.56 to 1.92). Specifically, pregnancy loss due to induced abortion and miscarriage instead of stillbirth increased CVD (HR 2.11, 95% CI 1.82 to 2.44, and 1.47, 95% CI 1.16 to 1.72, respectively). The risk of CVD gradually increased from ≤23 years to 23–25, 26–29 and ≥30 years with HR 1.29, 95% CI 1.24 to 1.34.

**Conclusion:**

Chinese women that have experienced pregnancy loss due to induced abortion and miscarriage had increased risk of CVD. The risk increased with the number of pregnancy losses and older age at the first pregnancy loss.

## Introduction

Cardiovascular diseases (CVDs) are a leading cause of death in China and the worldwide (1,2). Significant associations between pregnancy loss and risk of future maternal CVD have been found in Western countries (3,4,5). Pregnancy loss, a common adverse pregnancy outcome, is the death of a foetus at any time during pregnancy. It includes induced abortion, miscarriage and stillbirth. Globally, around a quarter of women have experienced an induced abortion (1), and 44 miscarriages occur each minute (6). There were about 2.6 million stillbirths in 2009 (7). Whilst the rate of pregnancy loss varies in countries depending on the religion, culture and policy (8), China has had a high occurrence of induced abortion between 1982 and 2015 due to one-child policy (9).

The current two studies performed in Chinese population had inconsistent findings in association between pregnancy loss and CVD (10). A large prospective cohort among textile workers in Shanghai did not find a significant association of spontaneous abortion, induced abortion and stillbirth with ischaemic heart disease, ischaemic stoke and haemorrhagic stroke (11). Whilst the data from the China Kadoorie Biobank (CKB) suggested that miscarriage, induced abortion and stillbirth were associated with adjusted HRs of 1.04 (1.01; 1.07), 1.04 (1.02; 1.07) and 1.07 (1.03; 1.11) for circulatory disease (12). Therefore, the role of pregnancy loss in CVD is still unclear in Chinese population. This study aimed to investigate the association of incidence of CVD with pregnancy loss, the number of pregnancies lost, subtypes of pregnancy loss (i.e. induced abortion, miscarriage and stillbirth) and age at first pregnancy loss using data of a nationwide cohort.

## Methods

### Study population

Data from the China Health and Retirement Longitudinal Study (CHARLS) were analysed. The China Health and Retirement Longitudinal Study is an ongoing nationally representative longitudinal survey of 17,708 people aged ≥45 years from 28 provinces in China (13). It was initiated in 2011 and had three follow-up visits in 2013, 2015 and 2018. Pregnancy history was additionally assessed using the one-off Life History Survey Questionnaire in 2014. The CHARLS protocol was approved by the ethical review committee at the Peking University and the Chinese Center for Disease Control and Prevention, and written informed consent was obtained from all participants (13). For this study, we included 7,495 women participants who did not have a history of CVD before the first pregnancy loss or the first birth.

### Measurement of pregnancy loss

Any occurrence and number of pregnancy losses, and details of each pregnancy loss (i.e., subtypes—induced abortion, miscarriage and still birth and age at the time) were recorded in the Life History Survey Questionnaire. Number of pregnancy losses was reclassified to three groups (i.e., 0, 1 and ≥2). Age at abortion was categorized based on the quartiles (i.e., ≤23, 23–25, 26–30, ≥30).

### Measurement of CVD

Coronary heart disease (CHD) included heart attack, coronary disease, angina and congestive heart failure. Women who had either a CHD or stroke was defined to have a CVD. The data CVD occurrence was obtained from the harmonized dataset, and the corresponding year of the occurrence was extracted from individual survey. The occurrence of CVD prior to CHARLS baseline visit was recorded via a question of ‘Have you been diagnosed with [the condition] by a doctor?’; and any occurrence of CVD between CHARLS visits was asked via a question of ‘Have you been diagnosed with [the condition] by a doctor [since Responder’s LAST interview MONTH, YEAR/ in the last two years]?’.

### Covariates

#### Basic information

At baseline in 2011, trained interviewers collected information on sociodemographic status and health-related factors using a structured questionnaire, including age, sex, coupleness and educational level. In this study, coupleness was reclassified into two groups: yes (i.e., married and partnered) and no (i.e., separated, divorced, widowed and never married). Educational level was reclassified as lower than primary school, equal to primary and secondary school and higher or equal to tertiary school. Self-reported health-related factors included smoking and drinking (i.e., have ever smoked and drank).

#### Physical information

Height and weight were measured by a trained nurse. BMI was calculated as weight in kilograms dividing by the square of height in meters (kg/m2). According to the standard classification of general adiposity measured by the BMI specific for Chinese adults, participants were categorized as follows: normal weight (<22.9 kg/m2), overweight (23.0–24.9 kg/m2) and obese (≥25.0 kg/m^2^) (14). Comorbidities included self-reported physician-diagnosed diabetes and hypertension conditions. Physical activity (PA) was assessed by the International Physical Activity Questionnaire with the question for level of activities (i.e., vigorous, moderate and walking for at least 10 min continuously in a normal week) (15). The amount of PA score per day was indexed based on the time spent on each type of PA, and the detailed calculation rules were presented in a previous study (16). Depression was assessed by the 10-item Center for Epidemiological Studies Depression Scale (17). A cut-off score of ≥10 was used to identify possible depressive symptoms (18).

#### Pregnancy information

Age at first birth, age at menarche, age at menopause and number of live births were recorded in the Life History Survey in 2014.

### Statistical analysis

Mean (standard deviation (SD)) and n (%) were used to describe baseline characteristics, split by any pregnancy loss (yes/no). Differences in baseline characteristics between women with and without pregnancy loss were examined by t tests for continuous variables and by χ^2^-test for categorical variables.

Multivariable-adjusted Cox proportional hazards regression models were constructed to examine the associations of pregnancy loss, number of pregnancy losses, subtype of pregnancy loss and age at first pregnancy loss with incident CVD in all, and CHD and stroke respectively. Hazard ratios (HRs) and 95% confidence intervals (CIs) of CVD, CHD and stroke were computed. The follow-up time of the study members were calculated from age at first pregnancy loss, or age at first birth if no pregnancy loss was reported, to age at first diagnosis of CVD, the end of follow-up, or loss to follow-up, whichever came first. Except for an univariable model (Model 1), a multivariable-adjusted model was built for adjusting of age at baseline of CHARLS, age at first birth or first pregnancy loss, menarche, menopause, number of live births, BMI categories, diabetes, hypertension, coupleness, smoking status, drinking status, education level, depression symptom and PA (Model 2). The analysis for the association between subtype of pregnancy loss and CVD was restricted to women who had no or one pregnancy loss, in which no pregnancy loss was the reference group.

Multiple imputation by chained equations was used to address missing data of those co-variates (0.1%–7.5% missing) assuming missing at random, with 20 imputations performed using completed and non-missing variables (19). Complete-case analyses were performed as a sensitivity analysis. To evaluate the long-term effect of pregnancy loss on CVD risk, we conducted a sensitivity analysis by excluding participants who had a CVD 10 years within their first pregnancy or pregnancy loss. Moreover, the interactions between subtype of pregnancy loss and the number of pregnancy losses as well as the age at first loss was evaluated for the risk of CVD. The interaction between the number of pregnancy losses and the age at first loss was also assessed. For significant interactions, subgroup analyses would be conducted. All statistical analyses were 2-sided with the significance level set at *p*≤0.05. All analyses were performed using Stata 16.1 (StataCorp LP, TX, USA).

## Results

### Baseline characteristics

Table 1 represents the baseline characteristics of CHARLS participants split by any experience of pregnancy loss. A total of 1,850 (24.7%) women experienced at least one pregnancy loss, and they were younger at baseline, gave first birth at older age and had a higher BMI, higher education level and less PA. Women with history of pregnancy loss tended be exposed to alcohol and live with partners, had menarche at a younger age, more gravidity and fewer live birth and were less likely to have hypertension compared with women without pregnancy loss.

Of 1,850 women experiencing pregnancy loss, 1,358 (73.4%) had one, 362 (19.6%) had two and 130 (7.0%) had three or more times pregnancies lost. Among 1,358 women with one pregnancy loss, 822 (61.9%) were those with induced abortion, 378 (28.5%) miscarriages, 128 (9.6%) stillbirths and 30 (2.2%) did not report the subtype of pregnancy loss. The cause of induced abortion was attributed to family planning policy (502, 61.1%), do not want to have a child regardless of the foetal gender (231, 28.1%), foetal gender preference (5, 0.5%), unhealthy foetus indicated by ultrasound result (14, 1.7%) and others (69, 8.4%). There were 441 (23.9%), 337 (18.3%), 592 (32.0%) and 477 (25.8%) women who had experienced their first pregnancy loss at age ≤23, 23–25, 26–30, ≥30 years, respectively.

**Table 1:** Baseline characteristics of study participants by pregnancy loss.


	PREGNANCY LOSS		p

	NO (n = 5636)	YES (n = 1850)	

Age when enrolled in the cohort, years	58.6 (9.7)	56.9 (8.7)	<0.001

Age at first birth, years	22.8 (3.5)	23.4 (3.3)	<0.001

Body mass index, kg/m^2^			

Normal weight	1,973 (44.0%)	646 (39.8%)	0.01

Overweight	894 (19.9%)	354 (21.8%)	

Obesity	1,616 (36.1%)	622 (38.4%)	

Education level, %			

Lower than Primary	2510 (44.5%)	560 (30.3%)	<0.001

Primary and secondary	2013 (35.7%)	688 (37.2%)	

Tertiary education	1113 (19.7%)	602 (32.5%)	

Smoker, %	389 (7.9%)	107 (6.9%)	0.17

Alcohol drinker, %	675 (13.8%)	249 (16.0%)	0.03

Physical activity score	157.2 (112.6)	140.0 (100.8)	0.001

Hypertension, %	1,255 (30.2%)	326 (25.7%)	0.002

Diabetes, %	292 (6.0%)	111 (7.2%)	0.098

Marital status-with partners, %	4,176 (84.7%)	1,380 (88.1%)	0.001

Age at menarche, years	16.1 (2.0)	15.9 (2.0)	<0.001

Age at menopause, years	48.7 (4.5)	48.8 (4.3)	0.59

Depression, %	2,001 (42.8%)	643 (43.1%)	0.832

Gravidity	3.1 (1.6)	4.0 (1.6)	<0.001

Number of live births	3.1 (1.6)	2.6 (1.5)	<0.001

1	581 (10.3%)	401 (21.7%)	<0.001

2	1,837 (32.6%)	664 (35.9%)	

3	1,382 (24.5%)	373 (20.2%)	

4	864 (15.3%)	214 (11.6%)	

5	972 (17.3%)	198 (10.7%)	


Values are percentages for categorical variables and means and standard deviations for continuous variables. P-value refers the comparison between women with history of pregnancy loss and without pregnancy loss. Total counts may vary because of missing values.

### The association between pregnancy loss and CVD

Over 39 years follow-up of the cohort, 2,055 (27.5%) women developed CVD (1,759 CHD and 508 stroke). Women with pregnancy loss tended to have incident CVD at earlier age compared with those without pregnancy loss (36.3 ± 9.5 years vs 43.1 ± 10.5 years, p < 0.001 for CHD and 34.2 ± 10.3 years vs 41.1 ± 11.3 years, p < 0 .001 for stroke) in the univariable model. After adjusting for covariates, women with a history of pregnancy loss had a significantly increased risk of CVD compared to those without pregnancy loss (HR 1.73, 95% CI 1.56 to 1.92, p < 0.001, Table 2). The association was similar for CHD (HR 1.71, 95% CI 1.53 to 1.91, p < 0.001) and stroke (HR 1.78, 95% CI 1.44 to 2.19, p < 0.001).

**Table 2:** Hazard ratios and 95% confidence intervals for cardiovascular disease associated with pregnancy loss and number of pregnancy loss among 7486 women.


	CARDIOVASCULAR DISEASE		CORONARY HEART DISEASE		STROKE	
		
	HR (95%)	p	HR (95%)	p	HR (95%)	p

Model 1						

Pregnancy loss (ever vs never)	1.81 (1.64 to 2.00)	<0.001	1.80 (1.62 to 2.00)	<0.001	1.77 (1.44 to 2.17)	<0.001

Number of pregnancy loss						

No pregnancy loss	1.00		1.00		1.00	

1	1.83 (1.63 to 2.05)	<0.001	1.82 (1.62 to 2.06)	<0.001	1.81 (1.44 to 2.28)	<0.001

≥2	1.77 (1.50 to 2.10)	<0.001	1.74 (1.44 to 2.08)	<0.001	1.67 (1.18 to 2.36)	<0.001

Continuous number	1.38 (1.30 to 1.46)	<0.001	1.36 (1.28 to 1.45)	<0.001	1.35 (1.20 to 1.53)	<0.001

Model 2						

Pregnancy loss (ever vs never)	1.73 (1.56 to 1.92)	<0.001	1.71 (1.53 to 1.91)	<0.001	1.78 (1.44 to 2.19)	<0.001

Number of pregnancy loss						

No pregnancy loss	1.00		1.00		1.00	

1	1.76 (1.57 to 1.98)	<0.001	1.75 (1.55 to 1.98)	<0.001	1.84 (1.45 to 2.33)	<0.001

≥2	1.65 (1.39 to 1.96)	<0.001	1.61 (1.34 to 1.94)	<0.001	1.62 (1.14 to 2.31)	0.01

Continuous number	1.28 (1.20 to 1.35)	<0.001	1.26 (1.19 to 1.34)	<0.001	1.28 (1.15 to 1.43)	<0.001


HR, hazard ratio. Model 1 was the univariable model. Model 2 adjusted age at baseline, age at first birth, menarche, menopause, number of live births, BMI, diabetes, hypertension, marital status, smoking status, drinking status, education level, depression symptom and physical activity.

### Number of pregnancy losses and CVD

In the adjusted models, continuous number of pregnancy losses was associated with an increased risk of CVD, CHD and stroke with HR 1.28, 95% CI 1.20 to 1.35, HR 1.26, 95% CI 1.19 to 1.34, and HR 1.28, 95% 1.15 to 1.43, respectively (all p < 0.001), but the risk was similar in women who experienced one and two or more pregnancy loss (Table 2).

### Subtype of pregnancy loss and CVD

**Figure 1:**
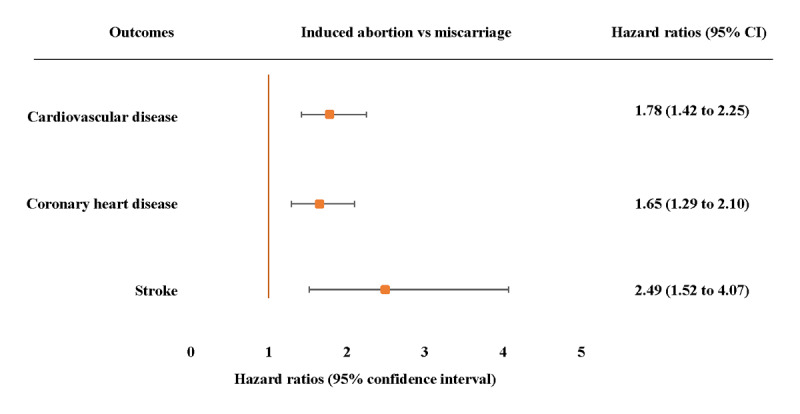
Adjusted hazard ratios (95% confidence intervals) for incident cardiovascular disease, coronary heart disease and stroke associated with induced abortion compared with miscarriage. Adjusted model 3 adjusted age at baseline, age at first birth, menarche, menopause, number of live births, BMI, diabetes, hypertension, marital status, smoking status, drinking status, education level depression symptom and physical activity. The hazard ratios (HRs) are plotted on a floating absolute scale. Each square has an area inversely proportional to the standard error of the log risk. Vertical lines indicate the corresponding 95% confidence intervals (CIs). Analyses are among women who had no or one pregnancy loss.

In the adjusted models, among women who experienced the pregnancy loss, we found that those who experienced an induced abortion had increased risk of CVD (HR 2.11, 95% CI 1.82 to 2.44), CHD (HR 2.02, 95% CI 1.73 to 2.36) and stroke (HR 2.70, 95% CI 2.02 to 3.62), those experienced miscarriage had increased risk of CVD (HR 1.41, 95% CI 1.16 to 1.72) and CHD (HR 1.47, 95% CI 1.19 to 1.81), but those who experienced a stillbirth did not have significantly increased risk of CVD, CHD or stroke (Table 3). Moreover, risks of CVD, CHD and stroke were higher for women who experienced induced abortion than those experienced miscarriage (Figure 1).

**Table 3:** Hazard ratios and 95% confidence intervals for cardiovascular disease associated with induced abortion, miscarriage and stillbirth among 6994 women.


	CARDIOVASCULAR DISEASE		CORONARY HEART DISEASE		STROKE	
		
	HR (95%)	p	HR (95%)	p	HR (95%)	p

Model 1						

No pregnancy loss	1.00		1.00		1.00	

Induced abortion	2.44 (2.12 to 2.81)	<0.001	2.36 (2.03 to 2.74)	<0.001	2.82 (2.13 to 3.75)	<0.001

Miscarriage	1.37 (1.13 to 1.67)	<0.001	1.43 (1.16 to 1.76)	<0.001	1.14 (0.74 to 1.74)	0.56

Stillbirth	1.11 (0.80 to 1.54)	0.52	1.09 (0.76 to 1.56)	0.64	1.10 (0.57 to 2.12)	0.79

Model 2						

No pregnancy loss	1.00		1.00		1.00	

Induced abortion	2.11 (1.82 to 2.44)	<0.001	2.02 (1.73 to 2.36)	<0.001	2.70 (2.02 to 3.62)	<0.001

Miscarriage	1.41 (1.16 to 1.72)	<0.001	1.47 (1.19 to 1.81)	<0.001	1.21 (0.78 to 1.86)	0.40

Stillbirth	1.29 (0.93 to 1.80)	0.13	1.28 (0.89 to 1.83)	0.18	1.28 (0.66 to 2.49)	0.47


This analysis was restricted to women who had no or one pregnancy loss. HR, hazard ratio. Model 1 was the univariable model. Model 2 adjusted age at baseline, age at first birth, menarche, menopause, number of live births, BMI, diabetes, hypertension, marital status, smoking status, drinking status, education level, depression symptom and physical activity.

### Age at first pregnancy loss and CVD

Age at the first pregnancy loss was positively associated with increased risk of CVD, CHD and stroke showing a ‘dose-response’ relationship (Table 4). The increased risk of CVD, CHD and stroke per increase in age group was significant (adjusted HR 1.29, 95% CI 1.24 to 1.34 CVD, HR 1.27, 95% CI 1.22 to 1.33 CHD, and HR 1.36, 95% 1.26 to 1.47 stroke, Table 4).

**Table 4:** Hazard ratios and 95% confidence intervals for cardiovascular disease associated with age at pregnancy loss among 7483 women.


	CARDIOVASCULAR DISEASE		CORONARY HEART DISEASE		STROKE	
		
AGE AT FIRST PREGNANCY LOSS	HR (95%)	p	HR (95%)	p	HR (95%)	p

Model 1						

No pregnancy loss	1.00		1.00		1.00	

≤23	1.20 (1.00 to 1.44)	0.048	1.22 (1.01 to 1.48)	0.041	1.14 (0.79 to 1.65)	0.48

23–25	2.03 (1.66 to 2.48)	<0.001	2.16 (1.75 to 2.67)	<0.001	1.26 (0.75 to 2.12)	0.38

26–29	2.16 (1.84 to 2.53)	<0.001	2.03 (1.71 to 2.41)	<0.001	2.63 (1.94 to 3.57)	<0.001

≥30	2.36 (1.98 to 2.82)	<0.001	2.27 (1.88 to 2.74)	<0.001	2.46 (1.71 to 3.52)	<0.001

Per 1 unit increase in age group	1.27 (1.23 to 1.31)	<0.001	1.26 (1.21 to 1.30)	<0.001	1.29 (1.20 to 1.38)	<0.001

Model 2						

No pregnancy loss	1.00		1.00		1.00	

≤23	1.14 (0.95 to 1.37)	0.15	1.15 (0.95 to 1.40)	0.15	1.11 (0.77 to 1.61)	0.58

23–25	1.75 (1.42 to 2.15)	<0.001	1.85 (1.49 to 2.30)	<0.001	1.15 (0.68 to 1.93)	0.61

26–29	1.96 (1.67 to 2.31)	<0.001	1.84 (1.54 to 2.19)	<0.001	2.62 (1.90 to 3.59)	<0.001

≥30	3.05 (2.54 to 3.67)	<0.001	2.89 (2.37 to 3.53)	<0.001	3.84 (2.62 to 5.63)	<0.001

Per 1 unit increase in age group	1.29 (1.24 to 1.34)	<0.001	1.27 (1.22 to 1.33)	<0.001	1.36 (1.26 to 1.47)	<0.001


HR, hazard ratio. Model 1 was the univariable model. Model 2 adjusted age at baseline, age at first birth, menarche, menopause, number of live births, BMI, diabetes, hypertension, marital status, smoking status, drinking status, education level, depression symptom and physical activity.

### Sensitivity and interaction analyses

After dropping women who had missing data in co-variates, there were 1,231 participants remained for analysis. The complete-case analyses did not substantially change the main results, except that the association between miscarriage and risk of CVD and CHD was no longer statistically significant in the fully adjusted model (Supplementary table 1). Sensitivity analyses by excluding participants whose CVD occurred within 10 years after their first pregnancy or pregnancy loss showed similar results compared to the full dataset (Supplementary table 2). There were no significant interactions between subtype of pregnancy loss and the number of pregnancy losses or the age at first loss, or between the number of pregnancy losses and the age at first loss, for the risk of CVD, CHD, and stroke (all p for interaction >0.2).

## Discussion

Using data from a nationwide longitudinal study in China, we evaluated the association between pregnancy loss and risk of future CVD in Chinese women. The results showed that pregnancy loss was associated with an increased risk of CVD. Importantly, similar risk of CVD was found in women who experienced one or more pregnancy loss. More importantly, we have identified that increased risk of CVD was from induced abortion and miscarriage, but not from stillbirth, and the risk was higher for induced abortion than miscarriage. There was a positive ‘dose-response’ relationship between age at first pregnancy loss and risk of CVD. The findings of our study have indicated that more attention should be paid to women who experienced pregnancy loss for the prevention of CVD, particularly in those who had induced abortion and miscarriage at an older age.

Many studies in Western populations have shown a significant association of pregnancy loss with CVD risk and mortality (3,5,20). However, the findings of studies conducted in Western countries might not be appliable to women in China because Chinese women have different characteristics from those western populations, e.g., low BMI, different patterns of dietary intake and a higher rate of induced abortion due to one child policy (9). Two studies from China have investigated the association of pregnancy loss with CVD risk (11,12), but the findings were not consistent. Our findings are principally consistent with those in a study from the CKB study (mean age 50.5 years) (12). The lack of a statistically significant association between stillbirth and risk of CHD and stroke in the present study compared with HR 1.07, 95% CI 1.03 to 1.11 for CHD and HR 1.06, 95% CI 1.01 to 1.12 for stroke in CKB study may be due to the small sample size of stillbirth in the CHARLS study. Similarly, the small sample size could contribute to the unsubstantial association between miscarriage and CVD in complete cases. However, the strength of association between pregnancy loss, including its number and induced abortion subtype, in our study was stronger than the CKB study and similar with the findings in Western populations. A possible reason may be that the study from CKB only used data of newly occurred CVD during the 7-year follow-up rather than all CVD since the first pregnancy loss or birth, and this could be supported by the similar results of sensitivity analysis excluding CVD occurred within 10 years after their first pregnancy or pregnancy loss (12). This suggests that the risk of CVD in women who experienced pregnancy loss may have been increased early in the reproductive lifespan.

Similar to the finding from the recent Nurses’ Health Study II, risk of CVD was increased with the number of pregnancy losses, but the risk was increased in women with two or more pregnancy losses compared with those with one pregnancy loss (HR 1.34, 95% CI 1.13–1.59 vs HR 1.18, 95% CI 1.06–1.31) (21), while we found a similar risk between two groups. This study is the first to determine the association of age at first pregnancy loss and risk of CVD in Chinese population. We found that the risk of CVD was higher in women at older age with pregnancy loss. This contrasts to the finding of the Nurses’ Health Study II which showed that the risk of CVD was greater for pregnancy occurring early in reproductive lifespan with HR 1.40 (95% CI 1.21–1.62) for age ≤23 years, and insubstantially increased risk for older age groups (21), moreover, the miscarriage occurring at early age increased the risk of death from CVD compared with that at older age (10). The reasons for differences in the impact of number and age of pregnancy loss on CVD risk between China and USA could be from the population characteristics, e.g., Chinese women had lower level of drinking and smoking, and also the differences in education level, public health and medical care, causes and subtypes of pregnancy loss (e.g. high proportion of induced abortion due to one child policy in China). The different impact of number and age at pregnancy loss on CVD needs to be considered for preventing CVD in later life in Chinese population.

Miscarriage and CVD shared similar risk factors, such as hypertension and diabetes (22,23). Vascular pathology including endothelial dysfunction, unbalanced hemodynamic and autoimmune disorder might result in the abnormal placentation for the pregnancy loss and CVD (24,25). Although we have adjusted for confounders including hypertension, diabetes and PA, further investigation of the underlying mechanism between miscarriage and CVD should be conducted.

### Strength and limitations

The China Health and Retirement Longitudinal Study is an ongoing nationally representative longitudinal survey of people from 28 provinces in China, which has wide social and geographic representation across China. Moreover, CHARLS includes the comprehensive measurement of variables, which enabled adjustment for well-known conventional risk factors in the Cox regression models. Several limitations are also acknowledged. There could be a recall bias for retrospectively obtained CVD at baseline. Our findings may not be generalisable to other populations, for example, Chinese women living in the western countries because of differences in lifestyles, environmental exposure and medical technology and care. There was a certain magnitude of missing data, but our results were verified by the sensitivity analysis. A large part of miscarriage was not brought to clinical attention, which might underestimate the number of miscarriage and attenuate the association with CVD.

### Implication of the study

Pregnancy loss as a risk factor of CVD in women has been included in the prevention of CVD in women in American College of Cardiology (ACC)/American Heart Association (AHA) guideline in 2020 (22), but the robust evidence on the association between pregnancy loss and maternal CVD in Chinese population is still lacking. This study provided supportive evidence to build relevant guidelines for Chinese population and inform both clinicians and women in reproductive age the potential hazard of induced abortion on future CVD. For prevention of CVD in Chinese women, pregnancy loss needs to be considered a risk factor in guideline of assessment, management or prevention of CVD in China.

## Conclusion

Pregnancy loss was associated with increased risk of future CVD in Chinese women. The association was stronger in those with older age pregnancy loss, and with pregnancy loss caused by induced abortion. The findings suggest that pregnancy loss had an undesirable influence on future cardiovascular health. This should be considered in the evaluation of CVD risk among women and be informed in obstetric clinic for future CVD prevention.

## Additional File

The additional file for this article can be found as follows:

10.5334/gh.1386.s1 Supplementary File. Supplementary tables 1 and 2.
