# Supplementary material for: Pregnancy Loss Was Associated With the Increased Risk of Cardiovascular Diseases in Middle-Aged Women: Evidence From the China Health and Retirement Longitudinal Study
Source: Glob Heart. 2025 Jan 9;20(1):1. doi: 10.5334/gh.1386 (PMC11720710; doi:10.5334/gh.1386)
Supplement: Supplementary File. — Supplementary tables 1 and 2. [file gh-20-1-1386-s1.pdf]

Supplementary table 1. Hazard ratios and 95% confidence intervals for cardiovascular disease associated with pregnancy loss (n=1231) in completed cases.

| | Cardiovascular disease | | Coronary heart disease | | Stroke | |
|---|---|---|---|---|---|---|
| | HR (95%) | p | HR (95%) | p | HR (95%) | p |
| ***Pregnancy loss (ever vs never)*** | 2.02 (1.59 to 2.55) | <0.001 | 1.87 (1.44 to 2.42) | <0.001 | 2.72 (1.72 to 4.30) | <0.001 |
| ***Number of pregnancy loss*** | | | | | | |
| No pregnancy loss | 1.00 | | 1.00 | | 1.00 | |
| 1 | 2.07 (1.58 to 2.71) | <0.001 | 1.96 (1.46 to 2.64) | <0.001 | 2.64 (1.57 to 4.44) | <0.001 |
| ≥2 | 1.90 (1.31 to 2.76) | <0.001 | 1.68 (1.11 to 2.54) | 0.01 | 2.90 (1.45 to 5.81) | <0.001 |
| Continuous number | 1.40 (1.23 to 1.61) | <0.001 | 1.31 (1.12 to 1.53) | <0.001 | 1.78 (1.39 to 2.28) | <0.001 |
| ***Subtype of pregnancy loss*** | | | | | | |
| No pregnancy loss | 1.00 | | 1.00 | | 1.00 | |
| Induced abortion | 2.67 (1.92 to 3.71) | <0.001 | 2.29 (1.59 to 3.29) | <0.001 | 4.89 (2.65 to 9.02) | <0.001 |
| Miscarriage | 1.25 (0.73 to 2.16) | 0.41 | 1.29 (0.72 to 2.31) | 0.40 | 1.43 (0.51 to 4.00) | 0.44 |
| Stillbirth | 1.69 (0.79 to 3.64) | 0.18 | 2.00 (0.93 to 4.32) | 0.08 | . | . |
| ***Age at pregnancy loss*** | | | | | | |
| No pregnancy loss | 1.00 | | 1.00 | | 1.00 | |
| ≤23 | 1.10 (0.71 to 1.71) | 0.67 | 1.02 (0.63 to 1.66) | 0.93 | 1.17 (0.69 to 1.99) | 0.55 |
| 23-25 | 2.10 (1.28 to 3.41) | <0.001 | 2.21 (1.31 to 3.73) | <0.001 | 1.23 (0.54 to 2.80) | 0.62 |
| 26-29 | 2.90 (2.05 to 4.12) | <0.001 | 2.56 (1.75 to 3.74) | <0.001 | 2.70 (1.66 to 4.39) | <0.001 |
| ≥30 | 3.40 (2.20 to 5.23) | <0.001 | 2.86 (1.76 to 4.66) | <0.001 | 5.14 (3.12 to 8.47) | <0.001 |
| Per increase in age group | 1.38 (1.27 to 1.51) | <0.001 | 1.33 (1.22 to 1.46) | <0.001 | 1.61 (1.36 to 1.90) | <0.001 |

HR, hazard ratio. Adjusted model adjusted age at baseline, age at first birth, menarche, menopause, number of live births, BMI, diabetes, hypertension, marital status, smoking status, drinking status, education level, depression symptom and physical activity.

Supplementary table 2. Hazard ratios and 95% confidence intervals for cardiovascular disease associated with pregnancy loss after excluding 2265 participants who had a CVD within 10 years of the first pregnancy (n=5221).

| | Cardiovascular disease | | Coronary heart disease | | Stroke | |
|---|---|---|---|---|---|---|
| | HR (95%) | p | HR (95%) | p | HR (95%) | p |
| ***Pregnancy loss (ever vs never)*** | 1.90 (1.64 to 2.21) | <0.001 | 1.92 (1.63 to 2.26) | <0.001 | 1.70 (1.26 to 2.30) | 0.001 |
| ***Number of pregnancy loss*** | | | | | | |
| No pregnancy loss | 1 | | 1.00 | | 1.00 | |
| 1 | 1.79 (1.50 to 2.13) | <0.001 | 1.78 (1.46 to 2.15) | <0.001 | 1.76 (1.24 to 2.50) | <0.001 |
| ≥2 | 2.18 (1.72 to 2.78) | <0.001 | 2.26 (1.75 to 2.91) | 0.01 | 1.57 (0.94 to 2.63) | 0.08 |
| Continuous number | 1.46 (1.33 to 1.59) | <0.001 | 1.47 (1.33 to 1.62) | <0.001 | 1.32 (1.10 to 1.59) | 0.003 |
| ***Subtype of pregnancy loss*** | | | | | | |
| No pregnancy loss | 1 | | 1 | | 1 | |
| Induced abortion | 2.54 (1.97 to 3.28) | <0.001 | 2.44 (1.85 to 3.21) | <0.001 | 3.20 (2.00 to 5.11) | <0.001 |
| Miscarriage | 1.42 (1.08 to 1.88) | 0.01 | 1.46 (1.08 to 1.98) | 0.01 | 1.23 (0.69 to 2.20) | 0.44 |
| Stillbirth | 1.31 (0.85 to 2.00) | 0.22 | 1.22 (0.75 to 1.99) | 0.41 | 1.07 (0.44 to 2.63) | 0.88 |
| ***Age at pregnancy loss*** | | | | | | |
| No pregnancy loss | 1 | | 1 | | 1 | |
| ≤23 | 1.35 (1.08 to 1.68) | 0.01 | 1.40 (1.11 to 1.78) | 0.005 | 1.18 (0.75 to 1.84) | 0.48 |
| 23-25 | 2.33 (1.69 to 3.19) | <0.001 | 2.49 (1.78 to 3.50) | <0.001 | 1.23 (0.55 to 2.80) | 0.61 |
| 26-29 | 2.48 (1.87 to 3.28) | <0.001 | 2.15 (1.56 to 2.96) | <0.001 | 3.98 (2.44 to 6.47) | <0.001 |
| ≥30 | 3.78 (2.75 to 5.20) | <0.001 | 3.72 (2.64 to 5.24) | <0.001 | 3.50 (1.67 to 7.35) | 0.001 |
| Per increase in age group | 1.39 (1.31 to 1.48) | <0.001 | 1.37 (1.28 to 1.46) | <0.001 | 1.42 (1.25 to 1.61) | <0.001 |

HR, hazard ratio. Adjusted model adjusted age at baseline, age at first birth, menarche, menopause, number of live births, BMI, diabetes, hypertension, marital status, smoking status, drinking status, education level, depression symptom and physical activity.
